# Development of a High-Throughput Microfluidic qPCR System for the Quantitative Determination of Quality-Relevant Bacteria in Cheese

**DOI:** 10.3389/fmicb.2020.619166

**Published:** 2021-01-07

**Authors:** Matthias Dreier, Hélène Berthoud, Noam Shani, Daniel Wechsler, Pilar Junier

**Affiliations:** ^1^Agroscope, Bern, Switzerland; ^2^Laboratory of Microbiology, University of Neuchâtel, Neuchâtel, Switzerland

**Keywords:** real-time qPCR, microbial community composition, microfluidic, cheese quality, cheese microbiome, fermented food, food microbiology, Fluidigm

## Abstract

The composition of the cheese microbiome has an important impact on the sensorial quality and safety of cheese. Therefore, much effort has been made to investigate the microbial community composition of cheese. Quantitative real-time polymerase chain reaction (qPCR) is a well-established method for detecting and quantifying bacteria. High-throughput qPCR (HT-qPCR) using microfluidics brings further advantages by providing fast results and by decreasing the cost per sample. We have developed a HT-qPCR approach for the rapid and cost-efficient quantification of microbial species in cheese by designing qPCR assays targeting 24 species/subspecies commonly found in cheese. Primer pairs were evaluated on the Biomark (Fluidigm) microfluidic HT-qPCR system using DNA from single strains and from artificial mock communities. The qPCR assays worked efficiently under identical PCR conditions, and the validation showed satisfying inclusivity, exclusivity, and amplification efficiencies. Preliminary results obtained from the HT-qPCR analysis of DNA samples of model cheeses made with the addition of adjunct cultures confirmed the potential of the microfluidic HT-qPCR system to screen for selected bacterial species in the cheese microbiome. HT-qPCR data of DNA samples of two downgraded commercial cheeses showed that this approach provides valuable information that can help to identify the microbial origin of quality defects. This newly developed HT-qPCR system is a promising approach that will allow simultaneous monitoring of quality-relevant species in fermented foods with high bacterial diversity, thereby opening up new perspectives for the control and assurance of high product quality.

## Introduction

Cheese can be considered a complex ecosystem that is characterized by multiple interactions between its diverse microbial community and environmental conditions. The cheese rind exhibits a high microbial diversity, whereas the composition of the microbiome within the cheese body is less complex (Wolfe et al., 2014; Dugat-Bony et al., 2016). Although the microbiota of raw milk is diverse, several factors, such as pretreatment of the milk, the use of starters, and the thermal conditions applied during cheese making, strongly influence the initial composition of the cheese microbiome. Moreover, the harsh environmental conditions occurring during ripening favor the development of a characteristic ripening microbiota that is especially adapted to an environment characterized by limited levels of fermentable carbohydrates, acidic pH, elevated salt concentrations, and low temperatures (De Filippis et al., 2014; Gobbetti et al., 2018).

The study of the bacterial community composition and of the bacterial population dynamics in cheese has been greatly improved with the advent of culture-independent molecular techniques. Methods such as denaturing gradient gel electrophoresis (DGGE), temporal temperature gradient gel electrophoresis (TTGE), single strand conformational polymorphism (SSCP), length heterogeneity PCR (LH-PCR), and terminal restriction fragment length polymorphism (T-RFLP), were commonly used in the past decades to study the microbial composition of raw milk and cheese, as well as the rind microbiota (Quigley et al., 2011). However, the recent development of next-generation sequencing (NGS) techniques has enabled an even more detailed study of complex microbiomes, and these are now the most widely used approaches in food microbial ecology (De Filippis et al., 2017). The commonest NGS technique used in the analysis of food microbiomes is 16S rRNA gene amplicon-based sequencing, which provides an extensive overview of the food microbiota (Cao et al., 2017). However, identification beyond the genus level is often not possible with this method (Claesson et al., 2010). In the case of cheese and dairy products, species level classification has been achieved by optimization of primer pairs for variable 16S rRNA gene regions, by improved data analysis procedures, and by the establishment of high-quality databases, such as the manually curated DAIRYdb (Meola et al., 2019).

Even though NGS is now routinely used by academic researchers, its use in the food industry is rare. An inherent limitation of the 16S rRNA gene sequencing method is that it only provides the relative abundances of the individual members of the community [operational taxonomic units (OTUs), amplicon sequence variants (ASVs), and taxa]. Complementary approaches, such as quantitative real-time PCR (qPCR) or flow cytometry, are then required to assess the quantitative aspects of the communities (Props et al., 2017). This quantitative analysis is particularly important for fermented foods, as off-flavors may arise due to the abundance of certain microbial populations (Giraffa, 2004). The composition of the cheese microbiome has an important impact on the sensory quality and safety of the final cheese product (Fox et al., 2017). The sensorial quality depends on the microbial biodiversity as well as on the bacterial counts of each individual species (Giraffa, 2004). The metabolic activity of desired and undesired bacterial species is usually sensorially perceivable at counts of >10^5^ colony-forming units per gram (CFU/g); however, easily noticeable flavor characteristics and off-flavors are typically associated with bacterial counts of 10^6^–10^9^ CFU/g (Fox et al., 2017).

Quantitative real-time PCR is a well-established method for the detection and quantification of bacteria, such as in pathogen detection in clinical and veterinary diagnostics and in food safety (Curran et al., 2007; Ramirez et al., 2009; Cremonesi et al., 2014; Sartori et al., 2017; Garrido-Maestu et al., 2018). The major limitation of standard qPCR methods is their low throughput, but this has been overcome in recent years with the development of high-throughput qPCR (HT-qPCR) platforms (Ishii et al., 2013; Waseem et al., 2019). HT-qPCR has now been validated and applied to investigate synthetic bacterial soil communities (Kleyer et al., 2017), to determine functional genes in soils (Crane et al., 2018), to quantify pathogens in spiked fecal and environmental water samples (Ishii et al., 2013), to study the gut microbial diversity in piglets (Hermann-Bank et al., 2013), and to quantify dairy *Lactococcus* (*Lc*.) *lactis* and *Leuconostoc* species bacteriophages (Muhammed et al., 2017). However, to our knowledge, HT-qPCR has not yet been used to quantify bacteria in fermented foods, such as cheese. Particularly in the case of raw milk cheeses, microbially induced quality defects, such as off-flavors caused by faulty secondary fermentation or the formation of high quantities of biogenic amines, can frequently lead to a downgrading of cheeses, with significant financial losses. A cost-effective monitoring of desirable and undesirable microorganisms could therefore improve the surveillance of product quality and enable the identification of the causes of microbial cheese defects at an early stage of ripening.

The present study describes the design, validation, and application of a novel microfluidic HT-qPCR system for the simultaneous quantification of multiple bacterial species that are frequently present in raw milk cheeses. We evaluated 24 qPCR assays targeting 23 different bacterial species, including two *Lactococcus lactis* subspecies. The selected target bacteria included lactic acid bacteria (LAB) often used as starters for cheese production, non-starter lactic acid bacteria (NSLAB), and selected species associated with undesired secondary fermentation. A workflow was also developed to facilitate the experimental setup, data filtering, and analysis of the HT-qPCR results. The developed HT-qPCR system was tested under practical conditions by inoculating experimental cheeses with different target species and by including two downgraded commercial cheeses with quality defects in the analysis.

## Materials and Methods

### Selection of Target Species and Primer Design

Twenty-four target species were selected based on a review of the literature and our own preliminary results from 16S rRNA gene amplicon-based sequencing of Gruyere and Raclette cheeses (unpublished data). The selection criteria were the abundance and frequency of detection, as well as known impacts on cheese quality (Table 1). The primer pairs used in this study are listed in Supplementary Table S1. New primer pairs were designed for 20 species according to the workflow described in a previous study (Dreier et al., 2020). Briefly, genome assemblies of the target species were downloaded from the National Center for Biotechnology Information (NCBI) and a pan-genome analysis was performed, single copy core genes were selected for primer design and species-specific primer pairs were identified. Three primer pairs were previously published (Dreier et al., 2020). LbhelvF1 and a modified version of LbhelvR1, described elsewhere (Moser et al., 2017), were selected as the primer pair for *Lactobacillus helveticus*. All primers were validated *in silico* by BLAST and Primer-BLAST searches (Johnson et al., 2008; Ye et al., 2012).

**TABLE 1:** Selected species/subspecies and their impact on cheese quality.

Species	Group	Associated defect	Incidence level
*Clostridium tyrobutyricum*	Raw milk contaminant	Butyric acid fermentation	Species
*Enterococcus durans*	NSLAB	Biogenic amines (T)	Species
*Enterococcus faecalis*	NSLAB	Biogenic amines (T)	Species
*Enterococcus faecium*	NSLAB	Biogenic amines (T)	Species
*Levilactobacillus brevis*	NSLAB	Biogenic amines (T)	Strain
*Lacticaseibacillus casei*	NSLAB	–	–
*Loigolactobacillus coryniformis*	NSLAB	Biogenic amines (H)	Strain
*Latilactobacillus curvatus*	NSLAB	Biogenic amines (T,P)	Strain
*Lactobacillus delbrueckii*	Starter	–	–
*Limosilactobacillus fermentum*	NSLAB/(Whey starter)	(Excess gas formation)	Species
*Lactobacillus helveticus*	Starter/Adjunct	–	–
*Lentilactobacillus parabuchneri*	NSLAB	Biogenic amines (H)	Strain
*Lacticaseibacillus paracasei*	NSLAB/Adjunct	–	–
*Lactiplantibacillus paraplantarum*	NSLAB	–	–
*Lactiplantibacillus plantarum*	NSLAB	–	–
*Lacticaseibacillus rhamnosus*	NSLAB/Adjunct	–	–
*Latilactobacillus sakei*	NSLAB	–	–
*Lactococcus lactis subsp. cremoris*	Starter	–	–
*Lactococcus lactis subsp. lactis*	Starter	–	–
*Leuconostoc mesenteroides*	Starter/Adjunct	–	–
*Pediococcus acidilactici*	NSLAB	–	–
*Pediococcus pentosaceus*	NSLAB	–	–
*Propionibacterium freudenreichii*	Adjunct/Raw milk	Propionic acid fermentation	Species
*Streptococcus thermophilus*	Starter	–	–

**T, Tyramine; P, Putrescine; H, Histamine.**

### Bacterial Strains

For each species, the type strain was selected; for additional strains, isolates from food were preferred. Strains (Supplementary Data Sheet 1, target and off-target strain sheets) were obtained from the Agroscope Culture Collection stored at −80°C in sterile reconstituted skim milk powder (10% w/v) and were reactivated and cultivated according to the conditions specified in Supplementary Data Sheet 1 (cultivation conditions sheet).

### DNA Extraction

DNA was extracted from bacterial single strains and from cheese samples, as follows. Bacterial pellets from single strains were harvested from 1 ml overnight cultures by centrifugation (10,000 × g, 5 min, room temperature). Bacterial pellets from cheese were obtained by adding 10 g of cheese to 90 ml modified peptone water (10 g/l peptone from casein, 5 g/l sodium chloride, 20 g/l trisodium citrate dihydrate, pH 7.0) and incubating for 10 min at 40°C. The sample was then homogenized for 3 min in a Stomacher (Masticator, IUL Instruments, Königswinter, Germany). A 50 μl volume of 10% (w/v) SDS was then added to 10 ml of the homogenate, which was then thoroughly mixed and centrifuged (4,000 × g, room temperature, 30 min). The bacterial pellets from the single strains and from the cheese samples were then subjected to a pre-lysis treatment, as described previously (Dreier et al., 2020). Briefly, the pre-lysis treatment included a 15 min incubation in 50 mM sodium hydroxide, followed by an incubation with 2.5 mg/ml lysozyme for 1 h at 37°C. Cell lysis and genomic DNA extraction was performed using the EZ1 DNA Tissue kit and a BioRobot^®^ EZ1 workstation (Qiagen, Hilden, Germany), according to the manufacturer’s instructions. Genomic DNA was eluted in a volume of 100 μl and the concentration was measured using a NanoDrop^®^ ND-1000 spectrophotometer (NanoDrop Technologies, Thermo Fisher Scientific, Waltham, MA, United States).

### Reagents and Conditions for Standard qPCR

The inclusivity of the primer pairs was assessed by performing qPCR with 2 ng DNA of 2–34 strains of the target species in technical duplicates (Supplementary Data Sheet 1, target strains). The qPCR assays were performed in a total reaction mix volume of 12 μl, containing 6 μl 2× SsoFast^TM^ EvaGreen^®^ Supermix with low ROX (Biorad, Cressier, Switzerland), 500 nM of forward and reverse primers, and 2 μl of DNA. The qPCR cycling conditions consisted in an initial denaturation at 95°C for 1 min, followed by 35 cycles of 95°C for 5 s and 60°C for 1 min. The melting curve analysis was performed using a gradient from 60 to 95°C, with 1°C steps per 3 s. All qPCR assays were run on a Corbett Rotor-Gene 3000 (Qiagen). Rotor-Gene 6000 Software 1.7 was used for analysis, with dynamic tube normalization and a threshold of 0.05 for quantification cycle (Cq) value calculation; the five first cycles were ignored for the determination of the Cq values. The peak calling threshold for the melt curve analysis was set to −2 dF/dT, and the temperature threshold was set at 2°C lower than the positive control peak.

### Preamplification of DNA Samples

An assay mix was prepared by pooling 1 μl of each primer (100 μM) in a total volume of 200 μl DNA suspension buffer [10 mM tris(hydroxymethyl)aminomethane, 0.1 mM ethylenediaminetetraacetic acid, pH 8]. A volume of 1.25 μl DNA sample was mixed with 3.75 μl preamplification pre-mix consisting of 2.5 μl 2× TaqMan PreAmp Master Mix (Thermo Fisher Scientific, Waltham, MA, United States), 0.5 μl of pooled assay mix, and 0.75 μl DNase-free water. Preamplification was performed using a Labcycler (SensoQuest, Göttingen, Germany) thermal cycler using the following conditions: an initial denaturation step at 95°C for 10 min, followed by 14 cycles at 95°C for 15 s and 60°C for 4 min. The preamplification primers were eliminated from the reactions by treating the samples with 2 μl diluted Exonuclease I (4 U/μl, Thermo Fisher Scientific, Waltham, MA, United States) at 37°C for 30 min, followed by enzyme inactivation at 80°C for 15 min. The final reactions were diluted 10-fold with DNA suspension buffer and stored at -20°C.

### Microfluidic HT-qPCR

HT-qPCR was performed using a 192.24 Dynamic Array integrated fluidic circuit (IFC; Fluidigm Corporation, San Francisco, CA, United States). DNA samples from pure bacterial cultures were diluted to 3 ng/μl prior to qPCR measurement. The assay mix consisted of 3 μl 2× Assay Loading Reagent (Fluidigm Corp.) added to 3 μl primer mix (forward and reverse, 10 μM). A sample pre-mix was prepared by combining 3 μl 2× SsoFast^TM^ EvaGreen^®^ Supermix with low ROX (Biorad, Cressier, Switzerland) and 0.3 μl 192.24 Delta Gene Sample Reagent (Fluidigm Corp.). Finally, 2.7 μl of each sample were added to 3.3 μl sample pre-mix. The IFC was loaded according to the manufacturer’s instructions (Fluidigm, 2015). Briefly, 3 μl of each assay and 3 μl of each sample were distributed to the respective inlet, and the IFC was loaded using the Juno Load Mix 192.24 GE script. The loaded IFC was transferred to the Biomark instrument and run with the GE 192x24 PCR+Melt v2 program, as follows: hot start 95°C for 1 min, followed by 30 cycles of denaturation at 96°C for 5 s and annealing and elongation at 60°C for 20 s. A melting curve analysis was performed with a temperature increase of 1°C per 3 s from 60 to 95°C.

### HT-qPCR Standards

**FIGURE 1:**
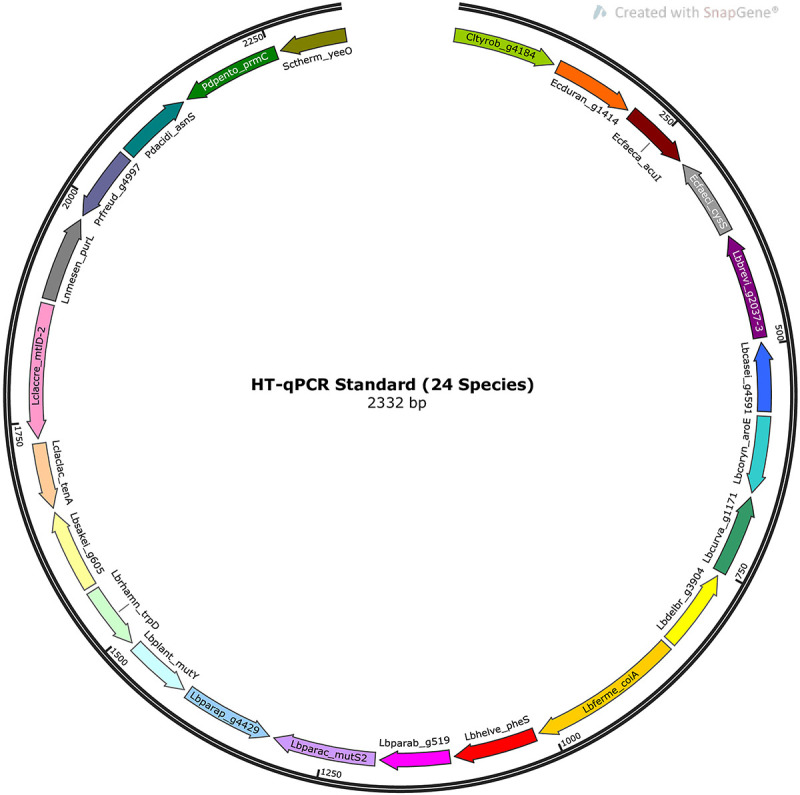
Map representation of the HT-qPCR standard. The standard is a linear, double-stranded DNA molecule 2,332 base pairs in length and consisting of target sequences of 24 species separated by thymine spacers 5 base pairs in length. The map was created using SnapGene Viewer 5.0.7.

The standards for quantification in the HT-qPCR system were produced using standard calibration curves of gBlock^TM^ Gene Fragments (Integrated DNA Technologies, LubioScience, Switzerland), consisting of 24 double stranded target species sequences separated by thymine spacers five base pairs in length. A map representation of the HT-qPCR standard is shown in Figure 1, and the sequence is available in Supplementary Data Sheet 2. The dried gBlock gene fragment pellet (Molecular weight: 1440635.7 u) was resuspended with DNA suspension buffer [10 mM tris(hydroxymethyl)aminomethane, 0.1 mM ethylenediaminetetraacetic acid, pH 8] at a concentration of 10 ng/μl. Copy numbers were calculated using the following equation:

$$10\,\frac{n\,g}{l}\times 0.69\,\frac{f\,m\,o\,l}{n\,g}\times 1\times 10^{-15}\,\frac{m\,o\,l}{f\,m\,o\,l}\times 6.022\times 10^{23}\,\frac{c\,o\,p\,i\,e\,s}{m\,o\,l}=4.16\times 10^{9}\,c\,o\,p\,i\,e\,s/l$$

### HT-qPCR Standard Calibration Curves

Copy numbers for quantification were calculated using duplicate standard calibration curves ranging from 10^8^ to 10^3^ copies/μl (Supplementary Figure S1 in Supplementary Data Sheet 3).

### HT-qPCR Samples

We assessed the specificity of the primer pairs using DNA from pure cultures of 84 strains (Supplementary Data Sheet 1, off-target strains sheet). For each strain, the cultivation from stock culture and the DNA extraction were performed twice independently. With the exception of *Leuconostoc mesenteroides* (four strains) and *Lacticaseibacillus casei* (two strains), three strains of each target species were selected. In addition, we also selected DNA of 12 type strains of species often occurring in dairy products or closely related to one of the target species (*Leuconostoc carnosum* and *Streptococcus salivarius*). The HT-qPCR was performed with DNA samples diluted to 3 ng/μl.

A mock community consisting of the type strains of the 24 target species/subspecies at concentrations of about 1 × 10^6^ copies/μl was also prepared (Supplementary Data Sheet 1, Mock community sheet). The DNA concentration for the corresponding number of genome copies was estimated by taking the genome size of the type strain, if available. Otherwise, we used the average genome size^1^ and an average weight of 1.096 × 10^–21^ g per base pair. A 10-fold dilution series of the mock community was prepared and subjected to preamplification to enrich the target sequences in the mock community dilutions (10^4^–10 copies/μl). Mock community dilutions without preamplification (10^5^–10^2^ copies/μl) were also measured.

### 192.24 Dynamic Array IFC Setup

The validation was performed on multiple 192.24 Dynamic Array IFCs. All samples (pure bacterial culture DNAs, no template controls, mock community, and HT-qPCR standard dilution series) were included, and eight primer pairs were measured in triplicate in each run.

### Production of Model Cheeses With Adjunct Cultures

Fifteen model cheeses with adjunct cultures of selected target species [*Levilactobacillus brevis, L. casei, Loigolactobacillus coryniformis, Latilactobacillus curvatus, Limosilactobacillus fermentum, L. helveticus, Lentilactobacillus parabuchneri, Lacticaseibacillus paracasei, Lactiplantibacillus plantarum, Lacticaseibacillus rhamnosus*, *Latilactobacillus sakei, Leuconostoc mesenteroides, Pediococcus* (*Pd*.) *acidilactici, Pd. pentosaceus*, and *Propionibacterium* (*Pr*.) *freudenreichii*] and 4 control cheeses (without adjunct cultures) were produced in the experimental cheese dairy at Agroscope (Bern, Switzerland) on four different days. The experimental design for the production of the 19 model cheeses and the conditions used for the preparation of the 15 adjunct cultures are listed in Supplementary Table S2. The pasteurized vat milk was inoculated by centrifuging 50 ml of each adjunct culture (4,000 × g, room temperature, 10 min) and resuspending in 50 ml sterile reconstituted skim milk powder (10% w/v) before addition to the milk. The estimated concentration of adjunct culture in the milk vat was 10^4^–10^5^ CFU/ml.

The Raclette-type semi-hard model cheeses were produced from 50 l of pasteurized milk, using a combination of the mesophilic starter RSW 901 (*Lc. lactis* ssp. *lactis*, *Lc. lactis* ssp. *cremoris*, *Lc. lactis* ssp. *diacetylactis*) and the mixed mesophilic/thermophilic starter MK 401 (*Lc. lactis* ssp. *lactis*, *Lactobacillus delbrueckii* subsp. *lactis* and *S. thermophilus*) (Liebefeld Kulturen AG, Switzerland). The milk was pre-ripened at 28–32°C for 30 min, followed by rennet addition and coagulation for 25 min at 32°C, and then cutting and stirring at 32°C for 25 min. The temperature was then increased to 36°C for 10 min and the milk was stirred for a further 35 min. The whey-curd mixture was filled into molds and pressed for 4 h at 34°C, 4 h at 32°C, and finally 8 h at 28°C. The cheeses (30 cm in diameter, about 6 kg) were immersed in a 20% (w/w) saline solution (11–13°C, 14 h), and smear-ripened in a maturing cellar (10–11°C, 90–96% relative humidity) for up to 120 days. The samples were collected after 111, 113, 118, and 120 days of ripening for the cheeses manufactured on days 1, 2, 3, and 4, respectively.

### HT-qPCR Application on Cheese Samples

We calculated the copy numbers for quantification using standard calibration curves ranging from 10^7^ to 10^3^ copies/μl (Supplementary Figure S2 in Supplementary Data Sheet 3). The measurement was performed on a single 192.24 Dynamic Array IFC. All samples (HT-qPCR standard dilutions, no template controls, and cheese samples) were measured in technical triplicates.

### Data Analysis

Results from the 192.24 Dynamic Array IFCs for the validation runs were combined for the analysis with the Fluidigm Real-Time PCR Analysis Software version 4.5.2 (Fluidigm Corp.). The quality threshold was set to 0.5, the quantification cycle (Cq) threshold for all reactions was set to 0.05, and the baseline correction was set to constant. The settings used for the melting curve analysis were: a peak sensitivity of 3 and a peak ratio threshold of 0.8, the qPCR assay-specific peak detection ranges are available in Supplementary Table S3. The melting curve peak threshold was set to 0.05 −dRn/dT for the validation runs and to 0.025 −dRn/dT for the run with the cheese samples, based on visual inspection of the baseline fluorescence. The Real-Time PCR Analysis Software flags all reactions that do not conform to the selected thresholds (i.e., low quality score, multiple or no melting curve peaks, or reactions where the normalized fluorescence is below the threshold). The data were then exported to a csv file. A python script (biomarkdataparser.py) was used to filter the data and to calculate the number of copies/μl in the samples based on the calibration curves. All reactions flagged by the Real-Time PCR Analysis Software were interpreted as negative results. The copies/μl of the specific targets were calculated for each reaction using the standard calibration curves, and all reactions below an 800 copies/μl cut-off were interpreted as negative, as recommended by the manufacturer (Fluidigm, 2018). Average copies/μl were only calculated if at least two of three reactions were positive; otherwise, the results were interpreted as negative. The raw data (csv export) from the Real-Time PCR Analysis Software, the biomarkdataparser.py script and the jupyter-notebooks used to make the figures are available in Supplementary Data Sheet 4 and on GitHub^2^.

### Analysis of Volatile Carboxylic Acids and Biogenic Amines

Volatile carboxylic acids in cheese were esterified with ethanol, and analyzed by gas chromatography as described by Fröhlich-Wyder et al. (2013) using a Hewlett Packard HP 6890 gas chromatograph (Agilent Technologies, Basel, Switzerland) equipped with a Hewlett Packard Ultra 2 cross linked phenyl methyl silicone fused silica capillary column (50 m, 0.32 mm, 0.52 mm) and a flame ionization detector (FID). Biogenic amines in cheese were analyzed as described by Ascone et al. (2017) using a UPLC system (UltiMate 3000 RS; Thermo Fisher Scientific, Reinach, Switzerland) equipped with a C18 column (Accucore C18: 2.6 mm, 150 × 4.6 mm; Thermo Fisher Scientific, Reinach, Switzerland). All measurements were carried out in duplicate.

## Results

### Specificity of the qPCR Assays

The inclusivity of the qPCR assays was assessed by performing standard qPCR with DNA from single strains of each target species (Supplementary Data Sheet 5). The inclusivity was 100% for all the tested qPCR assays (Table 2). The qPCR assay for *L. casei* was only tested with two *L. casei* strains, due to the limited availability of these strains in public strain collections. The *in silico* validation of the primer pair showed that all available genomes of *L. casei* and *L. zeae* contain a perfectly matching target sequence, in contrast to genomes of any other species of the *Lactobacillaceae* family (NCBI:txid33958) available in the NCBI Microbial Genomes BLAST database, including complete and draft genomes (as of July 2020; data not shown).

**TABLE 2:** Standard qPCR results of inclusivity assessment.

Species	Mean Cq	SD	Mean T_*m*_	SD	Inclusivity
*Clostridium tyrobutyricum*	14.32	1.05	80.02	0.14	25/25
*Enterococcus durans*	14.5	1.27	81.3	0.22	25/25
*Enterococcus faecalis*	14.55	0.71	75.5	0.0	22/22
*Enterococcus faecium*	11.95	0.53	80.8	0.11	25/25
*Levilactobacillus brevis*	14.65	2.21	82.55	0.11	18/18
*Lacticaseibacillus casei*	14.36	0.41	83.4	0.12	2/2
*Loigolactobacillus coryniformis*	12.95	0.4	83.19	0.16	19/19
*Latilactobacillus curvatus*	13.4	0.58	82.21	0.09	25/25
*Lactobacillus delbrueckii*	14.62	1.15	83.52	0.09	34/34
*Limosilactobacillus fermentum*	14.19	1.29	87.12	0.1	24/24
*Lactobacillus helveticus*	14.42	1.32	78.42	0.13	24/24
*Lentilactobacillus parabuchneri*	13.98	1.45	81.56	0.29	25/25
*Lacticaseibacillus paracasei*	13.88	1.06	84.34	0.23	21/21
*Lactiplantibacillus paraplantarum*	14.04	1.02	84.21	0.23	14/14
*Lactiplantibacillus plantarum*	13.86	0.67	76.94	0.1	24/24
*Lacticaseibacillus rhamnosus*	13.93	1.42	81.92	0.48	24/24
*Latilactobacillus sakei*	11.98	0.45	83.0	0.06	24/24
*Lactococcus lactis subsp. cremoris*	14.28	1.18	81.23	0.17	25/25
*Lactococcus lactis subsp. lactis*	14.53	0.87	80.02	0.2	25/25
*Leuconostoc mesenteroides*	13.68	1.14	82.28	0.32	23/23
*Pediococcus acidilactici*	11.61	0.46	78.89	0.22	20/20
*Pediococcus pentosaceus*	12.64	0.96	78.48	0.1	25/25
*Propionibacterium freudenreichii*	15.1	0.85	85.5	0.03	25/25
*Streptococcus thermophilus*	13.73	0.82	78.89	0.24	25/25

**Mean values and standard deviation (SD) of quantification cycle (Cq) and melting temperature (T_*m*_) of inclusivity assessment by standard qPCR for strains of the target species. Raw data is available in Supplementary Data Sheet 5.**

**FIGURE 2:**
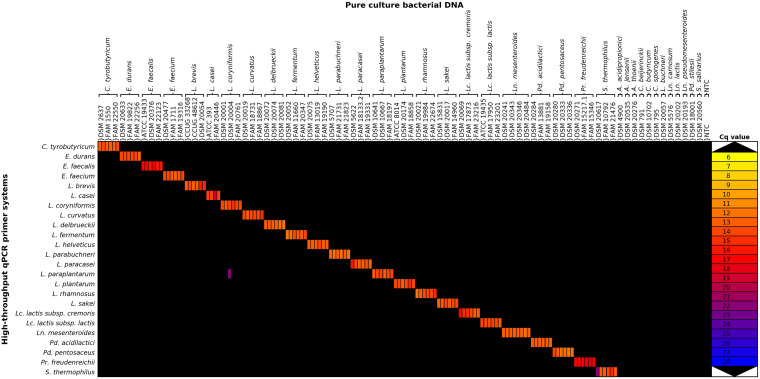
Heatmap of filtered average quantification cycle data of the exclusivity assessment. Average Cq values of the filtered Cq data are represented, but only data points where at least two of the three technical replicates were positive are depicted. Raw Cq data and filtered Cq data heatmaps are available in Supplementary Figures S3, S4 in Supplementary Data Sheet 3. *A*., *Acidipropionibacterium*; *C*., *Clostridium*; *E*., *Enterococcus*; *L*., *Lactobacillus*; *Lc*., *Lactococcus*; *Ln*., *Leuconostoc*; *Pd*., *Pediococcus*; *Pr.*, *Propionibacterium*; *S*., *Streptococcus*; NTC, no template control.

The specificity of the qPCR assays was assessed by performing HT-qPCR with DNA from single strains of two to four strains of the target and selected type strains of off-target species. The raw Cq data showed high quantification cycles for several off-target reactions, mainly for the qPCR assays for the detection of *L. delbrueckii*, *Lc. lactis subsp. lactis*, and *S. thermophilus* (Supplementary Figure S3 in Supplementary Data Sheet 3). Background noise was reduced by applying two filter criteria to the data: all reactions flagged by the analysis software and all reactions with fewer than 800 copies/μl were interpreted as negative reactions, as recommended by the manufacturer (Supplementary Figure S4 in Supplementary Data Sheet 3). All target species strains were detected by HT-qPCR, and only one cross-reaction was detected with the filtered average Cq values (Figure 2). The cross-reaction of the *Lactiplantibacillus paraplantarum* assay with the off-target strain *L. coryniformis* (DSM 20004) was only detected in one of two different DNA extracts, and the Cq value was about eight cycles higher than that for the *L. paraplantarum* DNA samples. In summary, the *L. paraplantarum* assay had a specificity of 0.9939, while all other tested assays were specific.

### Sensitivity and Dynamic Range of the qPCR Assays

The qPCR assay performance was assessed with a 10-fold dilution series of the qPCR standard consisting of all 24 target sequences in a range from 10^8^ to 10^3^ copies/μl. The calculated efficiency of the qPCR assays ranged between 87 and 97%. The linear regression equations (Cqslope*log[copies]*intercept*) had slopes between −3.39 and −3.68 and correlation coefficients between 0.992 and 0.998. The sensitivity of the assays without preamplification is given by the cut-off Cq value corresponding to 800 copies/μl (Cq 23.8–26.4), as calculated using the linear equations of the standard calibration curves (Supplementary Figure S1 in Supplementary Data Sheet 3).

**FIGURE 3:**
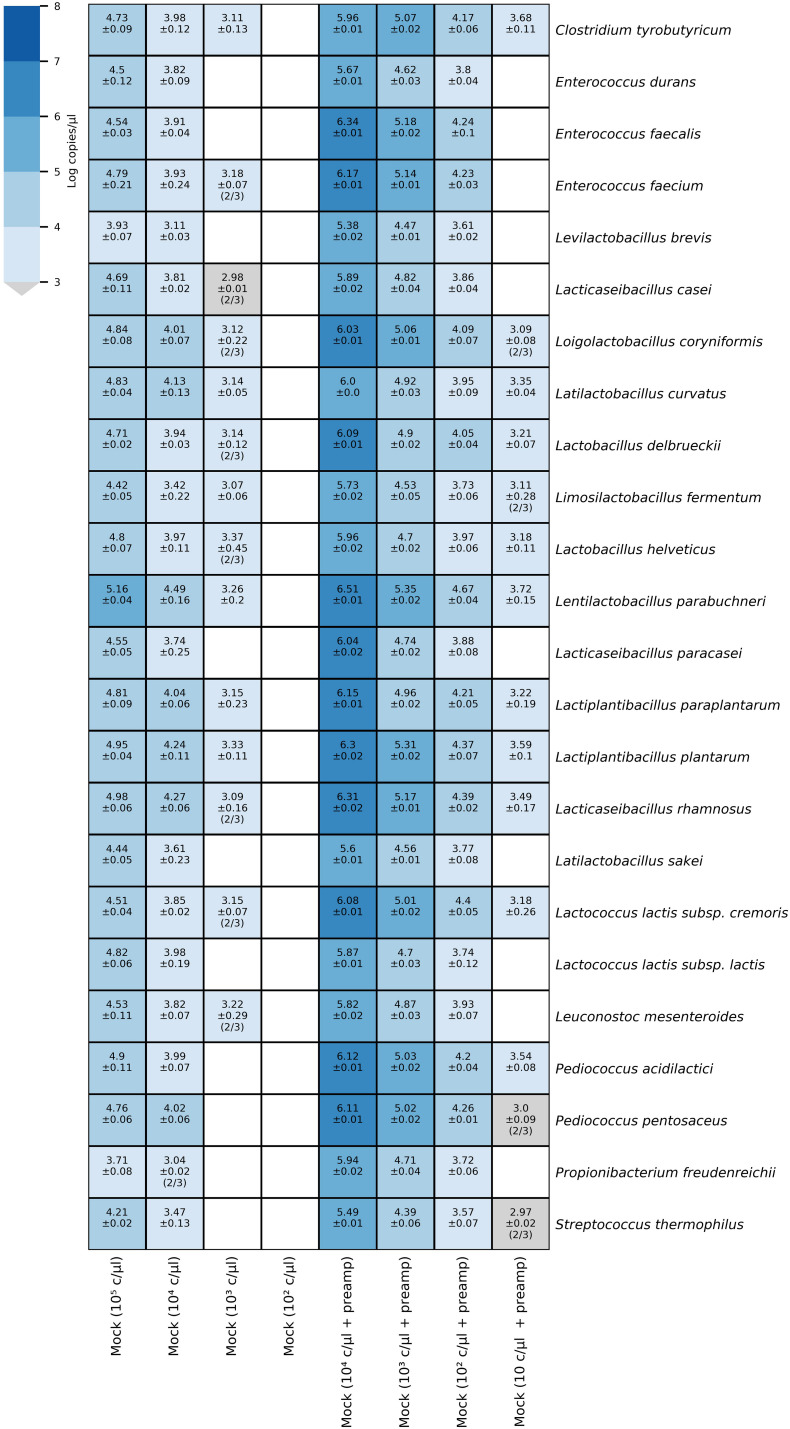
Heatmap of microfluidic qPCR results for mock community dilutions. The heatmap annotation depicts the average logarithmic copies/μl and the standard deviation. When not all samples were positive, the number of positive samples out of the total number of samples (triplicates) is given in brackets. On the left side, the results correspond to the diluted mock community DNA samples quantified without preamplification whereas, the diluted mock community DNA samples depicted on the right side were pre-amplified.

We validated the quantification of the targets in mixtures by HT-qPCR analysis of samples of a 10-fold dilution series of a mock community consisting of DNA from 24 type strains in a range between 10^5^ and 10^2^ copies/μl. All targets were detected in the diluted mock community sample containing 10^4^ copies/μl, and 14 of 24 assays detected the target a dilution of 10^3^ copies/μl (Figure 3). The concentrations of target DNA in the mock community were calculated based on the initial DNA concentration of the single strain sample and the genome size of the target species (Supplementary Data Sheet 1, Mock community sheet). The predicted concentrations were compared to the measured copies/μl (Supplementary Figure S5 in Supplementary Data Sheet 3). The assays for the detection of *L. brevis, L. sakei*, *L. paracasei*, *Pr. freudenreichii*, and *S. thermophilus* had lower initial concentrations of the target sequence than predicted. By contrast, the assays for *Enterococcus* (*E*.) *durans*, *E. faecalis*, *Lc. lactis subsp. lactis*, *Pd. acidilactici*, and *Pd. pentosaceus* showed similar values for the predicted and measured number of copies/μl, though the assays did not detect the target in the diluted sample containing 10^3^ copies/μl, whereas the 14 other assays did.

### Preamplification Efficiency

The increase in sensitivity due to preamplification reactions was assessed by preamplification of a 10-fold dilution series of a mock community consisting of DNA from 24 type strains in a range between 10^4^ and 10 copies/μl and subsequent HT-qPCR analysis. All species were detected down to a dilution of 10^2^ copies/μl in the pre-amplified mock community sample, whereas in the samples with the highest dilution of 10 copies/μl, 14 of 24 targets were detected (Figure 3). The efficiency of the preamplification reaction for the qPCR assays was assessed by comparing the Cq values obtained for a diluted mock community sample (10^4^ copies/μl) with preamplification to Cq values without preamplification (Table 3). The Cq values for the sample with preamplification decreased, on average, by 7.43 cycles (range 6.49–9.85) compared to the Cq values for the sample without preamplification.

**TABLE 3:** Quantification cycle values of a mock community sample with and without preamplification.

Species	Cq before preamplification	Cq after preamplification	ΔCq
*Clostridium tyrobutyricum*	20.71	13.67	7.05
*Enterococcus durans*	21.51	15.02	6.49
*Enterococcus faecalis*	22.89	14.43	8.45
*Enterococcus faecium*	21.10	13.32	7.78
*Levilactobacillus brevis*	23.42	15.51	7.90
*Lacticaseibacillus casei*	21.70	14.40	7.30
*Loigolactobacillus coryniformis*	20.65	13.56	7.09
*Latilactobacillus curvatus*	20.68	14.20	6.49
*Lactobacillus delbrueckii*	20.53	13.13	7.40
*Limosilactobacillus fermentum*	22.51	14.28	8.23
*Lactobacillus helveticus*	21.07	14.08	6.99
*Lentilactobacillus parabuchneri*	19.35	12.22	7.12
*Lacticaseibacillus paracasei*	21.21	13.24	7.97
*Lactiplantibacillus paraplantarum*	20.22	12.86	7.36
*Lactiplantibacillus plantarum*	20.96	13.60	7.36
*Lacticaseibacillus rhamnosus*	20.15	12.96	7.19
*Latilactobacillus sakei*	21.86	14.99	6.87
*Lactococcus lactis subsp. cremoris*	22.42	14.20	8.22
*Lactococcus lactis subsp. lactis*	20.85	14.32	6.53
*Leuconostoc mesenteroides*	21.00	13.96	7.04
*Pediococcus acidilactici*	20.55	13.15	7.40
*Pediococcus pentosaceus*	20.05	12.76	7.29
*Propionibacterium freudenreichii*	23.31	13.46	9.85
*Streptococcus thermophilus*	22.04	15.20	6.84

### Application of HT-qPCR to Raclette-Type Model Cheese

**FIGURE 4:**
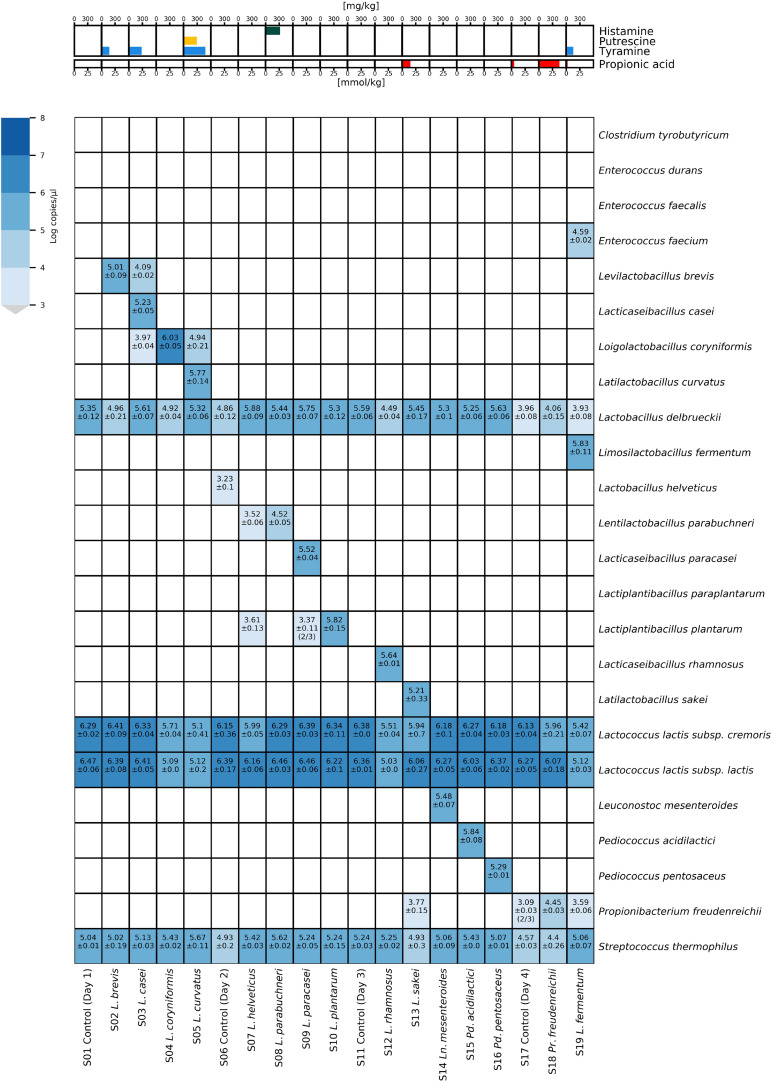
Heatmap of microfluidic HT-qPCR results of Raclette-type model cheese samples produced with adjunct cultures. The results of the chemical analysis of the model cheese are shown in the upper panel, while the results of the HT-qPCR analysis (without preamplification) are shown in a heatmap in the lower panel.

The ability of the HT-qPCR system to quantify the target species in real cheese DNA samples was verified by manufacturing 19 Raclette-type model cheeses with the target species adjuncts. The DNA extracts from 19 Raclette-type model cheeses were analyzed by HT-qPCR (Figure 4). The volatile carboxylic acids and biogenic amines of the cheeses were also analyzed, as these metabolites are often elevated in defective cheeses and serve as indicators of the presence of undesirable microorganisms.

The four starter LAB species (*Lc. lactis* subsp. *lactis*, *Lc. lactis* subsp. *cremoris*, *L*. *delbrueckii*, and *S. thermophilus*) were detected in all cheese DNA samples. All 15 adjunct culture species were detected in the corresponding cheese DNA sample, except for sample S07, where no *L. helveticus* was detected. Low concentrations of *L. helveticus* were detected in sample S06, indicating that the adjunct culture with *L. helveticus* had mistakenly been added to the wrong cheese vat. In several samples, cross-contaminations of target species from cheeses that were produced on the same production day were detected at distinct lower concentrations. The concentration of propionic acid was elevated in cheese samples S13 (14.93 mmol/kg) and S18 (37.83 mmol/kg) and, to a lesser extent, in cheese S17 (4.53 mmol/kg) and S19 (2.5 mmol/kg). Increased amounts of tyramine were measured in cheese samples S2 (171.78 mg/kg), S3 (284.67 mg/kg), S5 (482.33 mg/kg), and S19 (155.72 mg/kg), while in samples S5 and S8, the concentration of putrescine (292.8 mg/kg) and histamine (320.35 mg/kg) were increased, respectively.

### Application of HT-qPCR to Downgraded Commercial Cheeses With Quality Defects

**FIGURE 5:**
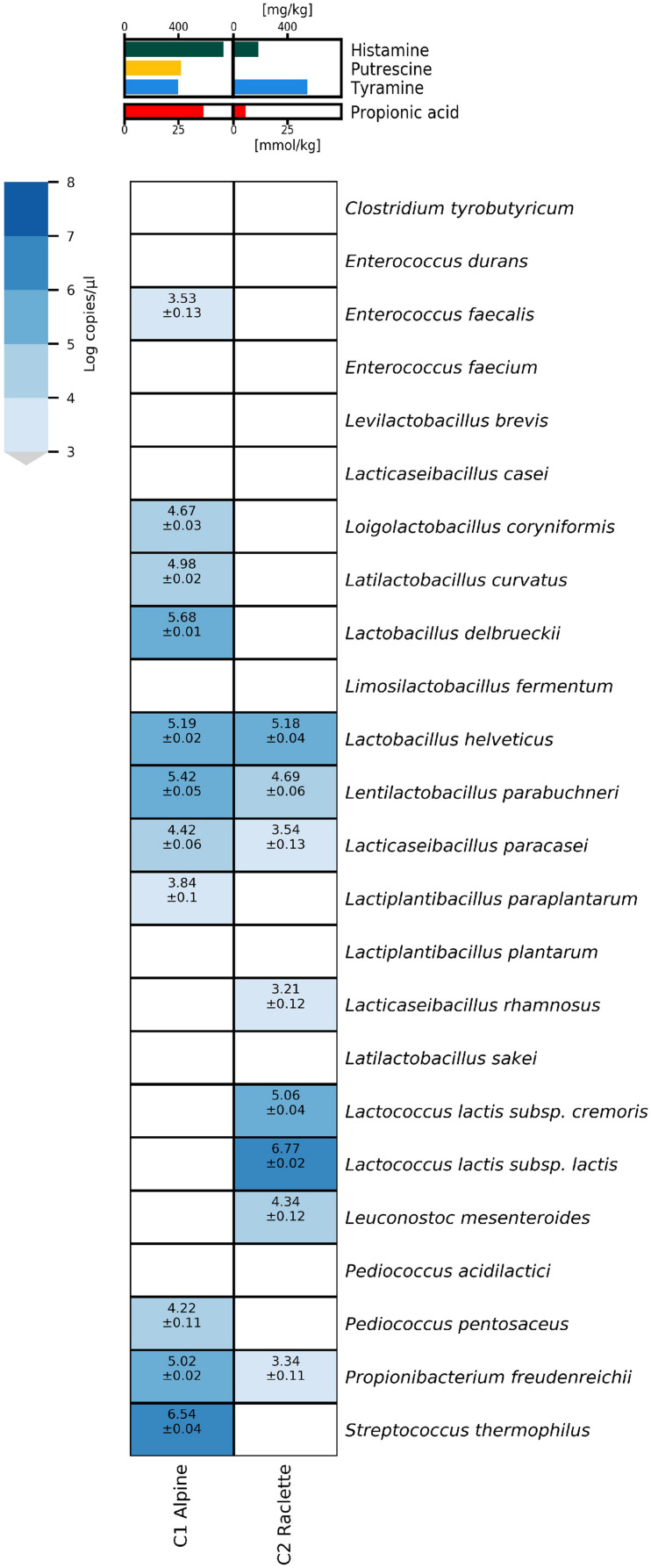
Heatmap of microfluidic qPCR results (without preamplification) for commercial cheese samples with defects.

The potential of the HT-qPCR system to identify the microbial causes of cheese defects was demonstrated by HT-qPCR analysis of DNA extracts from two commercial cheese samples with quality defects (the C1 alpine cheese and C2 Raclette cheese, Figure 5).

The alpine cheese sample (C1) had increased concentrations of propionic acid (36.5 mmol/kg) and biogenic amines, mainly histamine (733 mg/kg) and to a lesser extent tyramine (398 mg/kg) and putrescine (417 mg/kg). The cheese contained high concentrations of the typical thermophilic starter species *S. thermophilus*, whereas *L. delbrueckii. L. helveticus*, and *L. parabuchneri* were present at concentrations over 10^5^ copies/μl and *L. coryniformis*, *L. curvatus*, *L. paracasei*, and *Pd. pentosaceus* had concentrations between 10^4^ and 10^5^ copies/μl. Low concentrations (<10^4^ copies/μl) of *E. faecalis* and *L. paraplantarum* were detected.

The Raclette cheese sample (C2) had elevated levels of biogenic amines, mainly tyramine (545 mg/kg), but also histamine (185 mg/kg). Both subspecies of *Lc. lactis* and *Leuconostoc mesenteroides* used in mesophilic starters were detected, with *Lc. lactis subsp. lactis* as the predominant species at more than 10^6^ copies/μl. *L. helveticus* and *L. parabuchneri* were found at concentrations of about 10^5^ copies/μl, whereas *L. paracasei*, *L. rhamnosus*, and *Pr. freudenreichii* were present at concentrations below 10^4^ copies/μl.

## Discussion

### Validation of qPCR Assays and the Microfluidic HT-qPCR System

The qPCR assays validated in this study were highly specific. However, the qPCR assay for *L. casei* is not able to differentiate between *L. casei* and *L. zeae* species, two similar species for which a reclassification was recently proposed (Huang et al., 2020). The false positive cross-reaction of the *L. paraplantarum* assay in one *L. coryniformis* DNA sample was most likely due to a cross-contamination. A similar melting curve peak and the negative result of an independent DNA extraction from the same pure cultured strain support this assumption.

Background fluorescence signals in the raw data were mainly caused by three qPCR assays, specifically the assays for *L. delbrueckii*, *Lc. lactis* subsp. *lactis*, and *S. thermophilus*. Background signals may occur due to weak amplification of primer dimers in samples without the target sequence. The qPCR assay-specific cut-off values (equivalent to 800 copies/μl) were calculated from standard calibration curves and used to reduce background signals. Measures to increase the signal to the background ratio have also been reported in other microfluidic HT-qPCR studies. For example, a previous study (Ishii et al., 2013) reported that some (TaqMan) probes had to be redesigned because the probes failed to obtain sufficiently strong signals to separate them from background signals. Another study (Hermann-Bank et al., 2013) developed the Gut Microbiotassay on a 48 × 48 Access Array (Fluidigm Corp.) and excluded Cq values exceeding primer-specific cut-off values during data analysis.

The sensitivity of the tested qPCR assays was limited by the nanoliter-scale reactions used in the microfluidic qPCR system. This limitation for microfluidic HT-qPCR can be addressed by adding a preamplification step as a part of the experimental workflow. Preamplification increased the sensitivity of all assays in the mock communities. However, the delta Cq values calculated from target sequences and pre-amplified target sequences differed considerably for the 24 qPCR assays; consequently, the Cq data from samples with pre-amplification do not allow a reliable quantitative analysis and can therefore, only be used for qualitative detection of targets.

### Application of Microfluidic HT-qPCR to Cheese Samples

Given the technical limit of 800 copies/μl for qPCR reactions, the theoretical limit of detection of the assays was calculated as 8 × 10^4^ genome equivalents/g cheese. However, it should be noted that for culture-independent quantitative methods, the DNA extraction method can have a significant impact on the results obtained. It is known that residues from the food matrix such as fats, proteins and calcium in DNA samples can inhibit subsequent PCR reactions (Wilson, 1997). DNA extraction can also have an influence on the recovery rates of different bacteria, e.g., due to the different composition of cell walls and the resulting differences in the efficiency of cell lysis, such as between Gram-positive and Gram-negative bacteria (Quigley et al., 2012). Starter LAB grow very fast during cheese production, typically reaching counts of >10^8^ CFU/g within the first 24 h. By contrast, the growth of NSLAB is significantly slower and occurs mainly during the first weeks of ripening, reaching bacterial counts of 10^6^–10^8^ CFU/g, depending on the species (Fox et al., 2017). Quantitative studies have shown that the population density of species relevant for the organoleptic quality of cheese typically ranges from 10^6^ to 10^10^ genome equivalents/g cheese (Falentin et al., 2010, 2012; Turgay et al., 2011; Desfosses-Foucault et al., 2012; Moser et al., 2018). At lower population densities, the formation of metabolites is too low to be reliably perceived by sensory perception. The results obtained from the HT-qPCR analysis of the mock community dilutions indicate that all assays are able to quantify a minimal population density of 10^6^ genome equivalents/g cheese. Despite this rather high detection limit, HT-qPCR would still be a valuable tool for cost-effective monitoring of species relevant for the sensory quality of cheese. In addition, detection of species with lower abundancies (e.g., NSLAB species) in early stages of ripening could optionally be achieved using a preamplification step.

The application of the HT-qPCR system to model and commercial cheese samples was used to show the potential of the new method to detect a broad range of quality-relevant species in cheese samples, including starter LAB, NSLAB, and raw milk-associated contaminants that may cause severe cheese defects during ripening. The application of the microfluidic qPCR assays on model cheeses with adjunct cultures of selected target species confirmed the successful detection and quantification of these target species in cheese DNA samples. In addition, we observed the presence of bacteria that had not been deliberately added with the adjunct cultures in several cheese samples. These cross-contaminations most likely originated from equipment used in parallel during the simultaneous production of the experimental cheeses on the same day (e.g., cheese harps used for cutting the curd and the system used for filling the curd/whey mixture into the cheese molds). However, the unexpected presence of *Pr. freudenreichii* in sample S13 remains unexplained, as no adjunct culture with *Pr. freudenreichii* was used on that production day. The growth of *Pr. freudenreichii* in the cheese in sample S13 resulted in a similarly increased concentration of propionic acid (14.9 mmol/kg) as in other cheeses (S17, S18, and S19) in which *Pr. freudenreichii* was detected (Figure 4). Studies examining the environment and production facilities of cheese dairies show that bacteria present in raw milk and cheese are quite abundant and can persist on surfaces, despite frequent cleaning (Somers et al., 2001; Bokulich and Mills, 2013; Stellato et al., 2015). The source of the *E. faecium* contamination in S19 was identified as a contaminated stock culture of one of the used *L. fermentum* strains, as confirmed by partial 16S rRNA gene sequencing of single-colony DNA (Supplementary Data Sheet 6).

The selection of qPCR assays designed for the HT-qPCR system included species of undesirable bacteria found in raw milk. Various microbiologically induced quality defects in cheese are related to contamination of the processed milk with undesirable bacteria. The most common microbial causes of cheese defects are faulty fermentations, such as butyric acid fermentation (typically caused by *Clostridium tyrobutyricum*) and propionic acid fermentation (typically caused by *Pr. freudenreichii*), and the formation of biogenic amines (Bachmann et al., 2011). Tyramine, histamine, cadaverine, putrescine, and β*-*phenylethylamine (PEA) are the most abundant biogenic amines in cheese (Linares et al., 2011). Various NSLAB species play an important role in the excessive formation of biogenic amines in cheese (Barbieri et al., 2019). The formation of biogenic amines is a strain-specific characteristic of various NSLAB species. For example, strains of *L. parabuchneri* have been repeatedly isolated from cheeses heavily contaminated with histamine, whereas aminogenic strains of *E. faecium* are often present in cheeses with elevated tyramine content. Similarly, strains of *L. curvatus* have been shown to be potent producers of tyramine and putrescine (Benkerroum, 2016; Diaz et al., 2016; Wüthrich et al., 2017). The determination of metabolites like volatile carboxylic acids and biogenic amines often provides helpful information that clarifies the microbial origin of faulty fermentations and other cheese defects. However, the simultaneous quantitative determination of undesirable bacterial species using HT-qPCR opens up new perspectives for an efficient and cost-effective diagnosis of the causes of microbially induced cheese defects. Notably, the early and reliable detection of the microbial causes of cheese defects is an important precondition for tracing the sources of contamination and taking corrective actions.

In the model cheese experiments, samples with elevated tyramine content contained either *L. brevis*, *L. curvatus*, or *E. faecium*; all three species are known tyramine producers (Coton and Coton, 2009; Bunkova et al., 2010; Ladero et al., 2012). Sample S05 containing *L. curvatus* also showed elevated levels of putrescine, while sample S08 containing *L. parabuchneri* had elevated levels of histamine.

In the alpine cheese (sample C1), the histamine concentration was strongly increased (733 mg/kg), and an increased population density (5.42 log copies/μl) of *L. parabuchneri* was detected. The additional presence of *E. faecalis* and *L. curvatus* likely explains the formation of tyramine and putrescine. Moreover, the increased concentration of propionic acid correlates with the increased numbers of *Pr. freudenreichii* detected in this sample.

Similarly, the detection of *L. parabuchneri* most likely explains the increased concentration of histamine in the defective commercial Raclette cheese (sample C2). However, the results of the HT-qPCR analysis did not allow identification of a species that could account for the elevated tyramine content. In all likelihood, a species not covered by our qPCR assays was responsible for the high concentrations of tyramine. Strains of several *Lactobacillus* species other than the NSLAB species targeted here have been reported to produce tyramine (Bunkova et al., 2010; Benkerroum, 2016).

The setup of the method described here allows the exchange or extension of the qPCR assays for the detection of additional species or functional genes (e.g., the *hdc* gene, important in histamine production). Furthermore, the outlined workflow allows an efficient validation of new primer pairs for integration into the HT-qPCR system. We demonstrated here the potential of the HT-qPCR system to quantify simultaneously multiple bacterial species in cheese DNA samples. However, this approach could also be of interest for the investigation of other fermented foods such as kimchi, sauerkraut or sausages that also contain complex microbial compositions which include to some extent the same LAB species as present in cheese (Plengvidhya et al., 2007; Cocolin et al., 2009; Jung et al., 2011).

The HT-qPCR approach presented in this study offers a fast and affordable simultaneous quantitative screening of 24 species/subspecies relevant for the quality of cheese. A single 192.24 Dynamic Array IFC chip enables the screening of 56 cheese DNA samples in technical triplicates from 24 species/subspecies in several hours. Moreover, the developed script for data cleaning and visualization then allows immediate visualization and interpretation of the data exported from the Fluidigm Real-Time PCR Analysis software, thereby facilitating the rapid interpretation of the data. The high sample capacity of the microfluidic high-throughput system and the high specificity of the qPCR assays are key factors required for fast, accurate, and cost-efficient monitoring of desired and undesired microorganisms affecting sensory cheese quality.

Another advantage is that the system can easily be expanded with additional assays to cover further product-specific species or to adapt the system to other fermented products. Preliminary results from model cheeses and downgraded commercial cheeses showed that the application of HT-qPCR to complex fermented products such as cheese could be of interest for identification of the microbiological causes of sensorially perceivable quality defects. Particularly in the production of raw milk cheese, the application of HT-qPCR could be very useful for monitoring the composition of the ripening microbiota, thereby ensuring a constant product quality.

## Data Availability Statement

All datasets generated for this study are included in the article/Supplementary Material, further inquiries can be directed to the corresponding author.

## Author Contributions

MD, HB, NS, DW, and PJ conceived and designed the experiments, authored or reviewed drafts of the manuscript, and approved the final draft. MD performed the experiments, analyzed the data, and prepared figures and tables. All authors contributed to the article and approved the submitted version.

## Conflict of Interest

The authors declare that the research was conducted in the absence of any commercial or financial relationships that could be construed as a potential conflict of interest.

## Footnotes

1 https://www.ncbi.nlm.nih.gov/genome

2 https://github.com/biologger/htqpcr_validation_data

## Abbreviations

BLAST: basic local alignment tool
Cq: quantification cycle
DNA: deoxyribonucleic acid
*E*.: *Enterococcus*
HT-qPCR: high-throughput qPCR
*L*.: *Lactobacillus*
LAB: lactic acid bacteria
NSLAB: non-starter lactic acid bacteria
*Pd*.: *Pediococcus*
*Pr*.: *Propionibacterium*
qPCR: quantitative real-time polymerase chain reaction
*S*.: *Streptococcus*
Tm: melting temperature.
