# Supplementary material for: Development of a High-Throughput Microfluidic qPCR System for the Quantitative Determination of Quality-Relevant Bacteria in Cheese
Source: Front Microbiol. 2021 Jan 7;11:619166. doi: 10.3389/fmicb.2020.619166 (PMC7817891; doi:10.3389/fmicb.2020.619166)
Supplement: Supplementary file 3 [file Data_Sheet_3.docx]

Supplementary Material

# Supplementary Figures

**
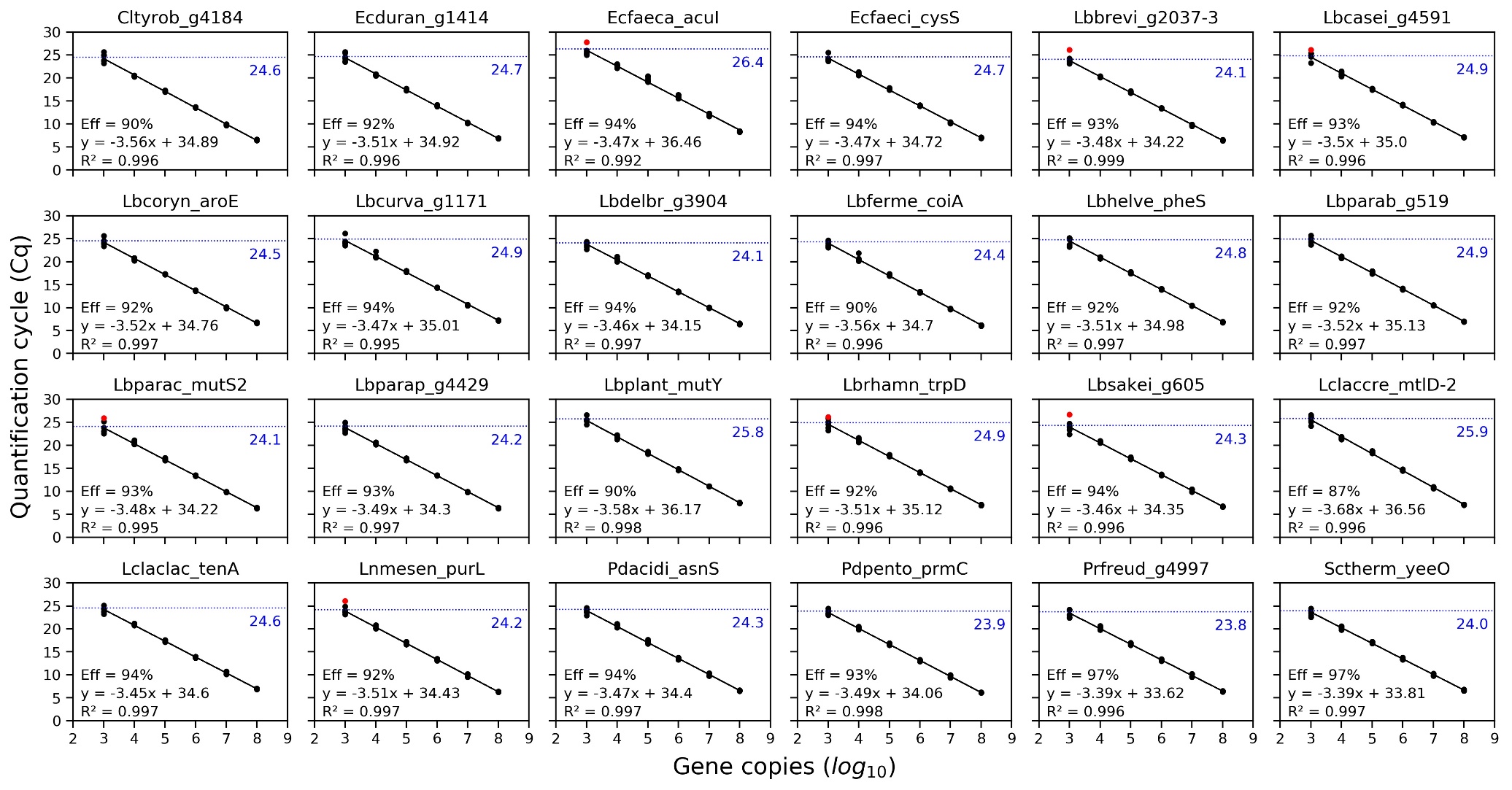
**

**Supplementary Figure S1**Standard calibration curves from tenfold dilution series of the HT-qPCR standard. Logarithmic copies/µl are plotted against the quantification cycles (Cq) of the species-specific primer pairs. Red dots represent data points not considered for the linear regression calculation, due to a low quality score (>0.5). The efficiency, linear regression equation, and correlation coefficients (R^2^) are shown in the lower left corner for each assay. The individual cut-off Cq values (corresponding to 800 copies/µl) are shown as a blue dotted line.

**
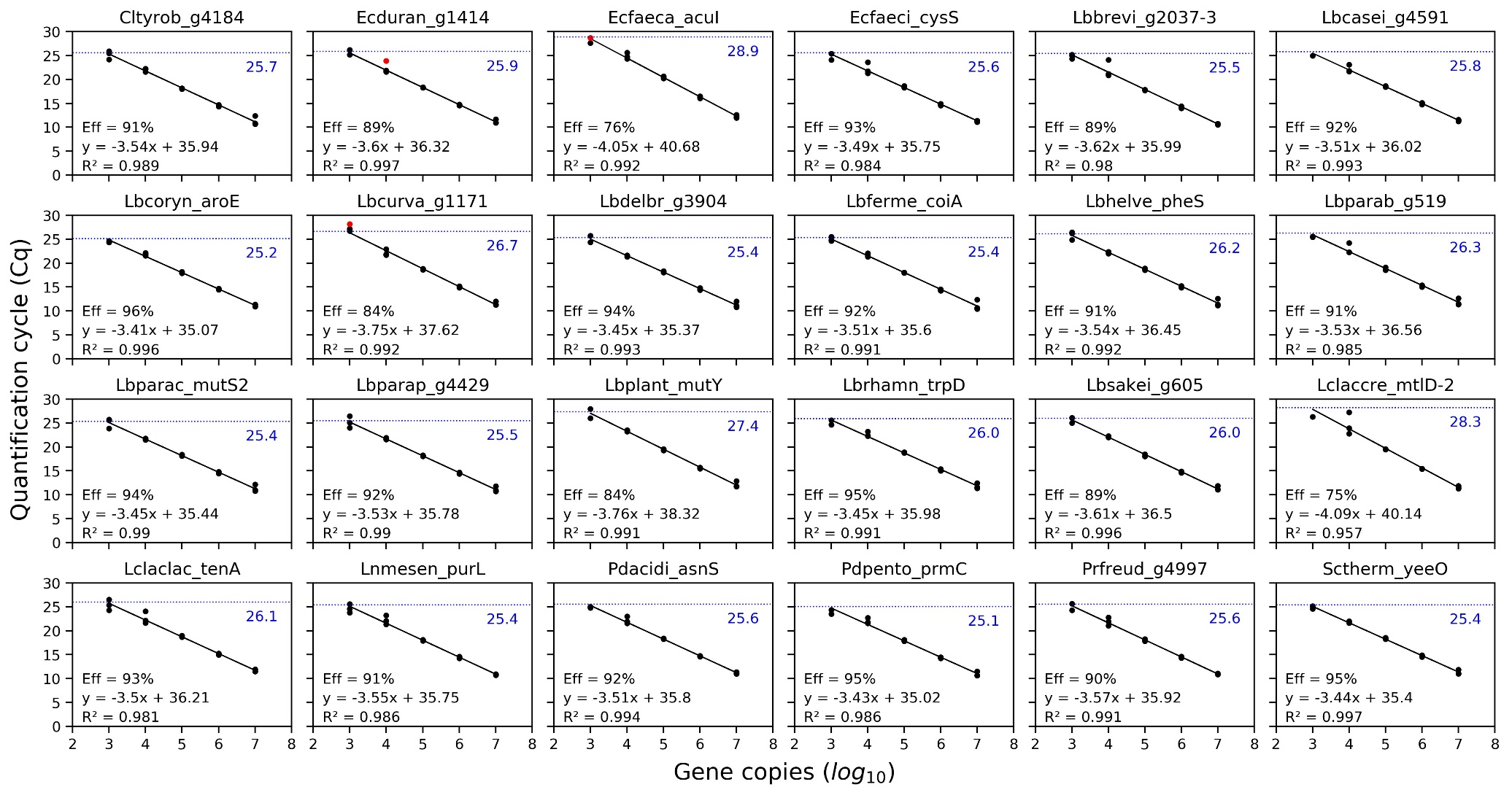
**

**Supplementary Figure S2**Standard calibration curves obtained from diluted series of the HT-qPCR standard used for quantification of bacterial target sequences in cheese samples. Logarithmic copies/µl are plotted against the quantification cycles (Cq) of the species-specific primer pairs. Red dots represent data points not considered for the linear regression calculation, due to a low quality score (>0.5). The efficiency, linear regression equation, and correlation coefficients (R^2^) are shown in the lower left corner for each assay. The individual cut-off Cq values (corresponding to 800 copies/µl) are shown as a blue dotted line.

**
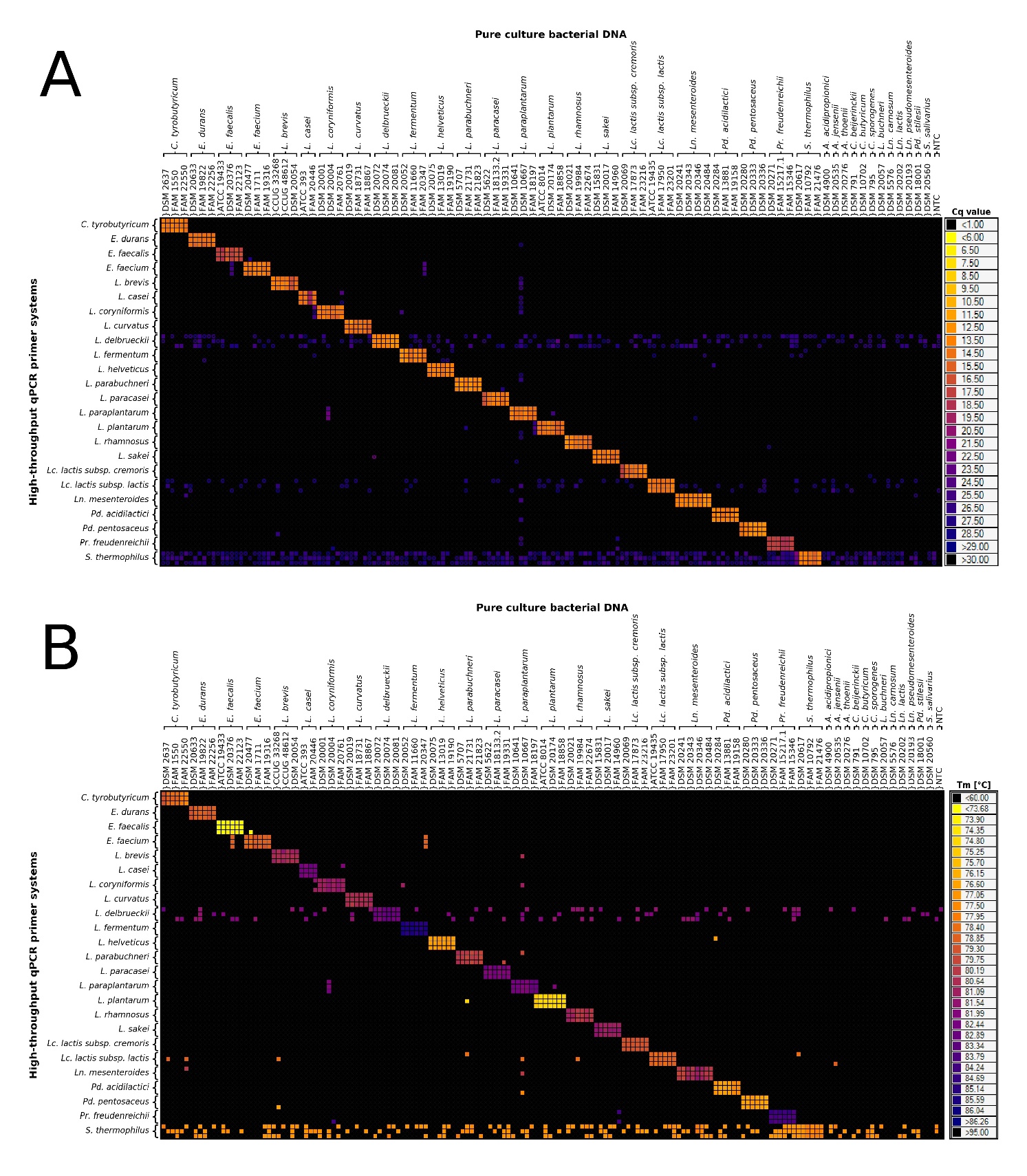
**

**Supplementary Figure S3**Heatmap of raw quantification cycle and melting curve analysis data. (A) Raw Cq heatmap generated by the Fluidigm Real-Time PCR Analysis Software. Data points that did not reach the quality threshold (0.5) or the melting curve peak threshold (0.05) are marked with an X in the heatmap. (B) Raw T_m_ (inside peak detection range) heatmap generated by the Fluidigm Real-Time PCR Analysis Software. Abbreviations: *A*., *Acidipropionibacterium*; *C*., *Clostridium*; *E*., *Enterococcus*; *L*., *Lactobacillus*; *Lc*., *Lactococcus*; *Ln*., *Leuconostoc*; *Pd*., *Pediococcus*; *Pr., Propionibacterium*; *S*., *Streptococcus*; NTC, no template control.


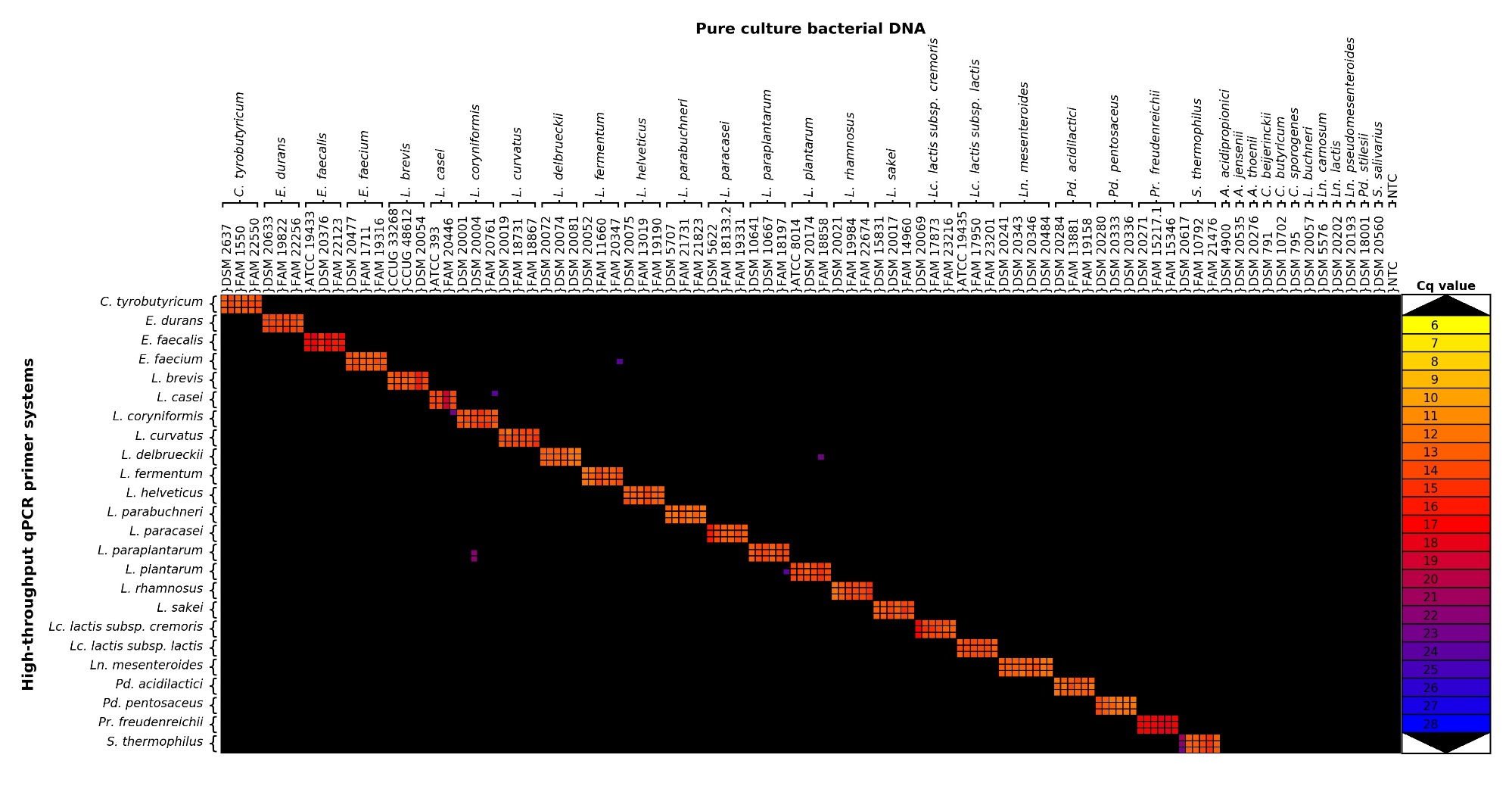


**Supplementary Figure S4**
Heatmap of filtered quantification cycle (Cq) data. Data cleaning was performed using the biomarkdataparser.py script. All flagged data points and data points with a Cq above the qPCR assay specific cut-off value were removed. Abbreviations: *A*., *Acidipropionibacterium*; *C*., *Clostridium*; *E*., *Enterococcus*; *L*., *Lactobacillus*; *Lc*., *Lactococcus*; *Ln*., *Leuconostoc*; *Pd*., *Pediococcus*; *Pr., Propionibacterium*; *S*., *Streptococcus*; NTC, no template control.

**
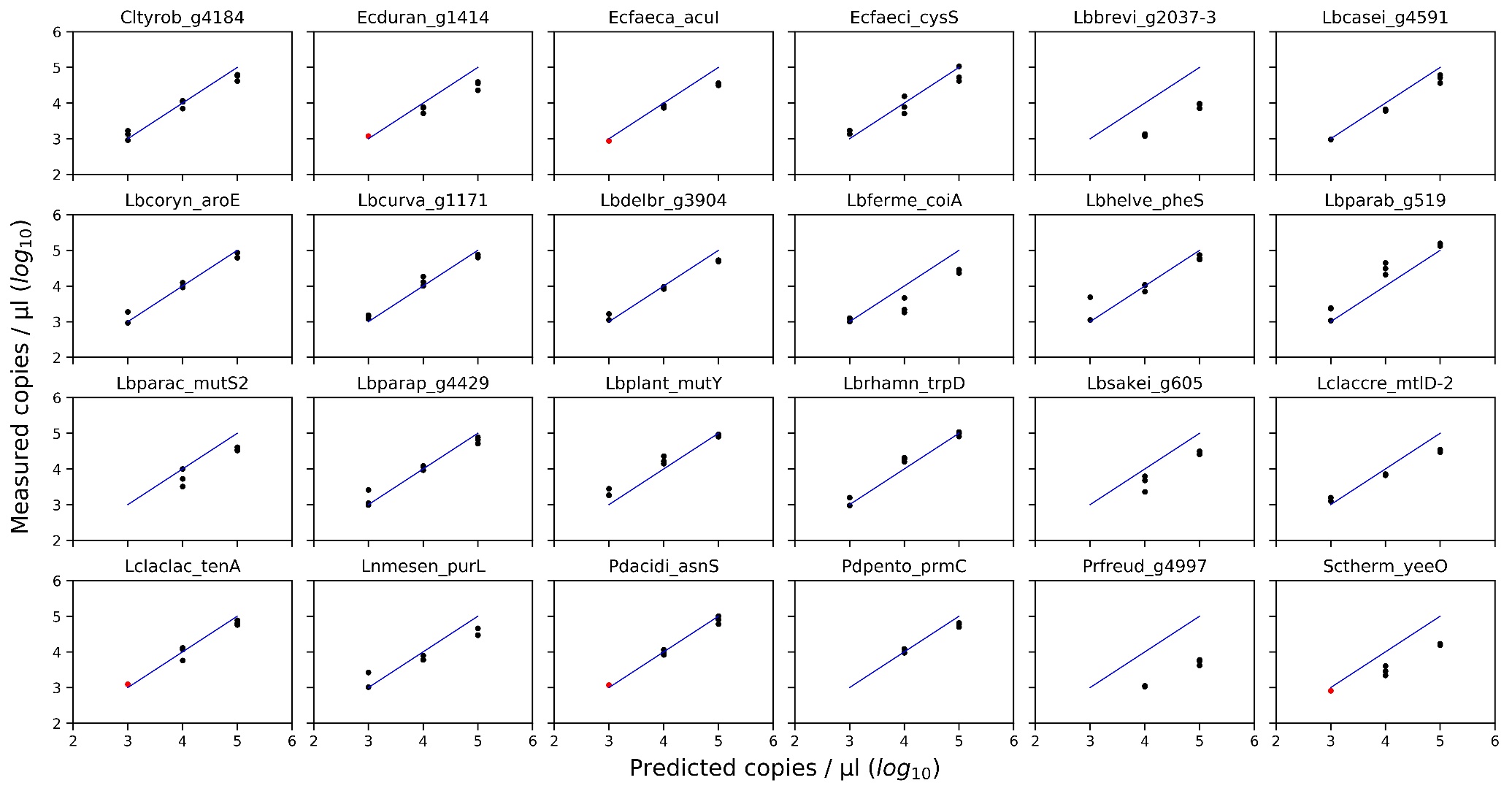
**

**Supplementary Figure S5**Plots of the predicted and measured population density of target species in diluted mock community samples without preamplification. Predicted logarithmic copies/µl were plotted against the measured logarithmic copies/µl. The blue line indicates perfect correlation. Red points represent single data points not considered in the average data in **Figure 3**.
