# Supplementary figures and images for: Development of a High-Throughput Microfluidic qPCR System for the Quantitative Determination of Quality-Relevant Bacteria in Cheese
Source: Front Microbiol. 2021 Jan 7;11:619166. doi: 10.3389/fmicb.2020.619166 (PMC7817891; doi:10.3389/fmicb.2020.619166)

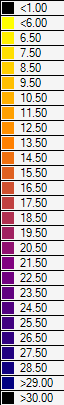

Supplement: Supplementary file 4 [file Data_Sheet_4.ZIP › htqpcr_validation_data-master/rawdata/bm_cq_legend.png]

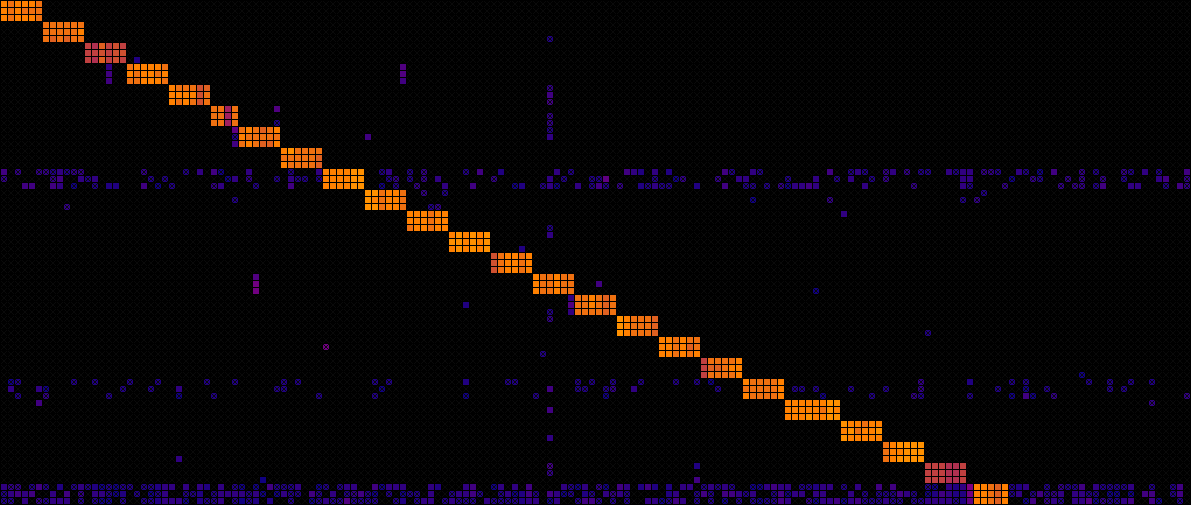

Supplement: Supplementary file 4 [file Data_Sheet_4.ZIP › htqpcr_validation_data-master/rawdata/bm_rawcqheatmap.png]

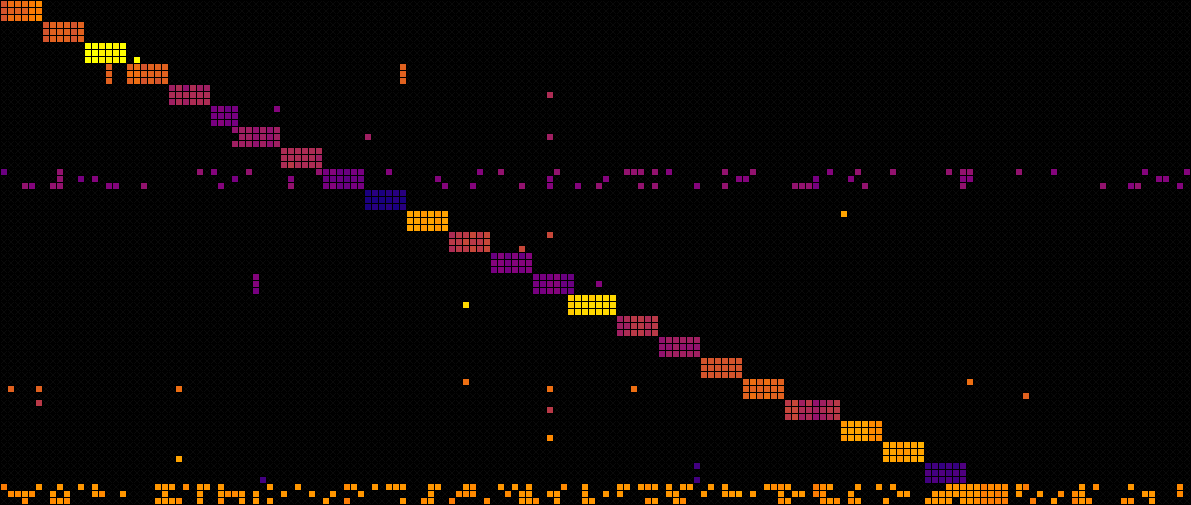

Supplement: Supplementary file 4 [file Data_Sheet_4.ZIP › htqpcr_validation_data-master/rawdata/bm_rawtmheatmap.png]

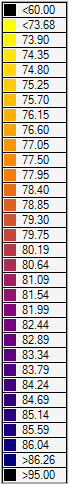

Supplement: Supplementary file 4 [file Data_Sheet_4.ZIP › htqpcr_validation_data-master/rawdata/bm_tm_legend.png]
