# Supplementary material for: Development of a High-Throughput Microfluidic qPCR System for the Quantitative Determination of Quality-Relevant Bacteria in Cheese
Source: Front Microbiol. 2021 Jan 7;11:619166. doi: 10.3389/fmicb.2020.619166 (PMC7817891; doi:10.3389/fmicb.2020.619166)
Supplement: Supplementary file 6 [file Data_Sheet_6.DOCX]

Supplementary Material:

Identification of the contaminant of the bacterial culture stock (FAM 20347).

# Material and Methods

## 16S rRNA gene amplification and sequencing

A part of the 16S rRNA gene was amplified with the primers 16SUNI-L and 16SUNI-R (Table 1) from the gDNA of the two isolates with different colony morphologies. The amplification was performed at a final volume of 25 ml, containing 200 nM of each primer, 2 U AmpliTaq Gold® Polymerase (Thermo Fisher Scientific, Waltham, MA, USA) 0.2 mM dNTPs, and 2.5 mL 10x PCR buffer I (Thermo Fisher Scientific, Waltham, MA, USA). The reactions were run on a TGradient thermocycler (Biometra, Göttingen, Germany) at 95 °C for 10 min followed by 35 cycles of 95 °C for 30 s, 54°C for 30 c, and 72 °C for 30 s, with a final extension for 7 min at 72 °C. The amplicon was purified using the MinElute PCR Purification kit (Qiagen, Hilden, Germany) according to the manufacturer’s protocol. The purified PCR products were sequenced by Sanger sequencing using the primer 16SUNI-L and 16SUNI-R, respectively (Fasteris SA, Plan-les Ouates, Switzerland).

Table 1: 16S rRNA gene primer used for amplification and sequencing

| **Primer** | **Sequence (5’-3’)** | **Reference** |
| --- | --- | --- |
| 16SUNI-L | AGA GTT TGA TCA TGG CTC AG | (Kuhnert et al., 1996) |
| 16SUNI-R | GTG TGA CGG GCG GTG TGT AC |  |

## BLAST search

An online BLAST search against the NCBI prokaryotic 16S ribosomal RNA database was performed with the sequences from Sanger sequencing.

# Results

The BLAST results of the search against the 16S ribosomal RNA database have shown that the PCR product of the gDNA from colony type 1 shows the highest similarity to the 16S rRNA sequence of *Lactobacillus fermentum* strain CIP 102980 and NBRC 15885 (Table 2). While the sequence of the second colony type shows the highest similarity to the 16S rRNA sequence of the *Enterococcus faecium* strains NBRC 100486, NBRC 100485 and ATCC 19434 (Table 3). In summary, the contaminant of the *Lactobacillus fermentum* strain FAM 20347 stock was identified as an *Enterococcus faecium* strain.

Table 2: BLAST results for the Query FAM20347.1_colony_type_1

| QUERY | FAM20347.1_colony_type_1 | | | | |
| --- | --- | --- | --- | --- | --- |
| BLAST HIT | **Species** | Query coverage [%] | Identity [%] | Sequence title | Accession |
| 1 | ***Lactobacillus fermentum*** | 99 | 99 | Lactobacillus fermentum strain CIP 102980 16S ribosomal RNA, partial sequence | NR_104927.1 |
| 2 | ***Lactobacillus fermentum*** | 99 | 99 | Lactobacillus fermentum strain NBRC 15885 16S ribosomal RNA gene, partial sequence | NR_113335.1 |
| 3 | ***Lactobacillus gorillae*** | 99 | 98 | Lactobacillus gorillae strain KZ01 16S ribosomal RNA, partial sequence | NR_134066.1 |
| 4 | ***Lactobacillus ingluviei*** | 99 | 96 | Lactobacillus ingluviei strain KR3 16S ribosomal RNA gene, partial sequence | NR_028810.1 |
| 5 | ***Lactobacillus gastricus*** | 99 | 95 | Lactobacillus gastricus strain Kx156A7 16S ribosomal RNA, partial sequence | NR_029084.1 |

Table 3: BLAST results for the Query FAM20347.1_colony_type_2

| QUERY | FAM20347.1_colony_type_2 | | | | |
| --- | --- | --- | --- | --- | --- |
| BLAST HIT | **Species** | Query coverage [%] | Identity [%] | Sequence title | Accession |
| 1 | ***Enterococcus faecium*** | 100 | 99 | Enterococcus faecium strain NBRC 100486 16S ribosomal RNA gene, partial sequence | NR_113904.1 |
| 2 | ***Enterococcus faecium*** | 100 | 99 | Enterococcus faecium strain NBRC 100485 16S ribosomal RNA gene, partial sequence | NR_113903.1 |
| 3 | ***Enterococcus faecium*** | 100 | 99 | Enterococcus faecium strain ATCC 19434 16S ribosomal RNA gene, partial sequence | NR_115764.1 |
| 4 | ***Enterococcus faecium*** | 100 | 99 | Enterococcus faecium strain DSM 20477 16S ribosomal RNA, partial sequence | NR_114742.1 |
| 5 | ***Enterococcus lactis*** | 99 | 99 | Enterococcus lactis strain BT159 16S ribosomal RNA gene, partial sequence | NR_117562.1 |

# References

Kuhnert, P., Capaul, S.E., Nicolet, J., and Frey, J. (1996). Phylogenetic positions of *Clostridium chauvoei* and *Clostridium septicum* based on 16S rRNA gene sequences. *Int J Syst Bacteriol* 46(4)**,** 1174-1176. doi: 10.1099/00207713-46-4-1174.
